# The relationship between depression, daytime napping, daytime dysfunction, and snoring in 0.5 million Chinese populations: exploring the effects of socio-economic status and age

**DOI:** 10.1186/s12889-018-5629-9

**Published:** 2018-06-19

**Authors:** Yuning Liu, Tingting Peng, Siqiao Zhang, Kun Tang

**Affiliations:** 1 School of Public Health, Peking University Health Science Center, Beijing, China; 2 Department of Global Health, Emory University Rollins School of Public Health, Atlanta, USA; 3 School of Public Health, Peking University Health Science Center, 38th Xueyuan Road, Haidian District, Beijing, China

**Keywords:** Daytime napping, Depression, Socio-economic status, Sleep duration, China

## Abstract

**Background:**

Daytime napping has been postulated as both a protective and a risk factor for depression in previous studies. In addition to these conflicting results, research gaps also exist with regard to controlling confounding bias between daytime napping and depression and examining the potential association within the Chinese population. To facilitate the prevention and diagnosis of depression, this study aims to provide insight into the association of daytime napping and depression in 0.5 million Chinese adults by fully controlling confounders, and further examine the modifying effects of socio-economic status (SES) and age.

**Methods:**

Data were drawn from the baseline of a Chinese cohort study of 0.5 million adults. Depressive status was measured by the Composite International Diagnostic Inventory (CIDI). Logistic regression models were used to examine the association between depression and daytime napping adjusted for SES, sleep-related factors, lifestyle factors and related diseases. Further stratified analyses were conducted to identify the modifying effects of socio-economic status and age.

**Results:**

The odds ratio of depression by daytime napping was 1.15 (95% CI: 1.01–1.31) in females and 1.42 (95% CI: 1.18–1.71) in males. Factors including living in a rural area (OR = 1.31, 95% CI: 1.13–1.52), receiving less education (OR = 1.42, 95% CI: 1.22–1.66), getting married (OR = 1.24, 95% CI: 1.10–1.40) and being 45–65 years old (OR = 1.29, 95% CI: 1.12–1.49) had a modifying effect on daytime napping and depression that could strengthen the association.

**Conclusions:**

A significantly positive association was found between depression and daytime napping, as well as daytime dysfunction, snoring and both shorter and longer sleep duration. Lower SES and age could possibly modify the association. Further clinical or epidemiological studies are needed to investigate the mechanism and facilitate the prevention of depression.

## Background

Over the past few decades, daytime napping has received the attention of many researchers [1–4]. 22–69% of older adults around the world take habitual daytime naps [5]. Especially in China, taking a nap after lunch is popular because culturally it is considered as a healthy lifestyle habit [6, 7]. However, the effects of daytime napping have remained uncertain for decades. Some studies have examined the positive influence of napping on memory consolidation, alertness improvements and well-being [8–10]. Other research has found that longer and more frequent naps were associated with increased morbidity and mortality among both elderly and youth populations [11, 12].

On the other hand, depression is a serious public health issue globally, and China is no exception [13, 14]. 37.9% of the Chinese population showing depressive symptoms and 4.1% of the population being diagnosed with clinical depression [13]. Evidence abounds as to the negative impact of depression on cardiovascular disease, memory performance, cognition and familial relationships [14–19]. However, less than 10% of depression patients in China were treated with antidepressant medications in 2016 [20]. More than 60% of antidepressant drugs were still imported without being included in the national essential drug list, which meant that most Chinese patients could not afford the cost of antidepressant drug treatment [20]. The development of accurate diagnosis and prevention of depression is crucial in improving the status of mental health in China.

Sleep and sleep disorders have been associated with depression in both clinical and population studies [21, 22]. The bidirectional relationship between insomnia and major depressive disorders is well established [23, 24]. Excessive daytime sleepiness (EDS), defined as the tendency to fall asleep during the daytime despite attempts to remain alert, has been established as a medical symptom of depression, and also a side effect of antidepressant treatment [25, 26]. Furthermore, obstructive sleep apnea (OSA), featured as snoring, fatigue and EDS, has been discovered having positive association with depression in clinical experiments and longitudinal population studies [22, 27].

Although the association of depression with sleep patterns has been assessed extensively in numerous studies, few studies have targeted on daytime napping specifically [28, 29]. The general reasoning for this lack of focus might be attributed to the challenges involved in controlling confounding bias. In terms of the relationship between daytime napping and depression, apart from basic socio-demographic status and lifestyle factors, there are four other major types of confounders which exist. Firstly, there is increasing evidences that sleep-related factors such as EDS, OSA, short sleep duration and poor sleep quality are positively related to depression, as mentioned previously [21, 22]. Daytime napping might be a manifestation of these sleep-related factors [30]. Secondly, a series of diseases including cardiovascular disease, hypertension, cancer, diabetes, stroke, kidney disease, asthma, cirrhosis, hepatitis, and rheumatoid arthritis have also been linked with both depression and daytime napping habits [28, 31–37]. Thirdly, specific lifestyle factors including physical activity or obesity may affect daytime napping habits significantly. These factors have also been associated with individual depressive episodes [38, 39]. Lastly, somnolence and insomnia, which might cause daytime napping behavior, have been proven to be side effects of antidepressant therapy [40].

In addition to controlling confounding bias, other research gaps regarding daytime napping exist. Due to limited studies, considerable uncertainty remains about the association between depression and daytime napping among Chinese population, whose daytime napping behaviour is prevalent and representative of other populations [41–43]. In addition, the modifying effect of socio-economic status (SES) still remains unknown, despite previous studies which found that SES was highly correlated with both depression and sleeping patterns, respectively [44, 45].

This study was conducted based on detailed analyses of the baseline data of a population-based cohort study of 0.5 million people selected from 10 regions in China. To address the research gaps, this study aimed to explore the true association between daytime napping and depression among Chinese population. Multiple operations were implemented to control related confounders to ensure reliable association. The modifying effect of SES and age were also examined.

## Methods

### Study design and participants

Data was requested from the baseline of a Chinese population-based cohort study conducted during 2004–2008. This large-sample prospective study aimed to provide a reliable assessment of the complex interplay of lifestyle, environmental and genetic factors as determinants of chronic disease. Further information of the baseline study has been described elsewhere [46]. A total of 512,891 adults (210,259 [41%] male, average age 52.9 years [SD = 10.90] and 302,632 [59%] female, average age 50.96 years [SD = 10.48]) 30–79 years old were recruited from 1737 communities among five rural and five urban regions of China. Participants in each administrative unit (rural villages or urban residential committees) selected for the study within each region were identified through official residential records [46]. Basic information regarding the participants, including demographic and socio-economic characteristics, lifestyle risk factors, as well as disease status was collected by well-trained healthcare professionals with electronic questionnaires. Blood sample analysis and physical examinations were conducted in medical centres.

This study was approved by the Institutional Review Board at Oxford University and the Chinese National Center for Disease Control. All participants were provided with informed consent and were allowed to withdraw at any time during the study.

### Exposures and outcomes

In this study, participants were asked, “Do you usually take a daytime nap?” Respondents who answered “yes” were assigned to the group of having a daytime nap, and respondents who answered “no” were assigned to the non-napping group.

The present study assesses depression by diagnosis using the Chinese version of the computerised Composite International Diagnostic Inventory-short form (CIDI-SF). All the participants were given a list of questions regarding their mental health condition (i.e., feeling sad, loss of appetite, loss of interest in activities once enjoyed, sense of worthlessness or uselessness) which lasted for two or more weeks during the previous 12 months. Respondents who answered one yes or more were then asked to complete the CIDI-SF at the assessment centre [47]. According to the WHO, depression is defined as a common mental disorder, characterised by sadness, loss of interest or pleasure, feelings of guilt or low self-worth, disturbed sleep or appetite, feelings of tiredness and poor concentration [48]. Major Depression (MD) was assessed with the result of this interview using the CIDI-SF. CIDI has been recognised having moderate concordance with clinical psychiatric interviews and reliability in diagnosis by the Diagnostic and Statistical Manual of Mental Disorders-IV (DSM-IV) [49]. In this study, MD was indicated by the presence of dysphoria and/or anhedonia. Anhedonia was accompanied by a clustering of somatic, cognitive and behavioural disturbances, including changes in appetite, weight, sleeping problems, feelings of guilt or worthlessness, psychomotor changes, fatigue, concentration problems and thoughts of suicide that lasted 2 weeks or more [50].

### Other covariates

Demographic and socio-economic characteristics and lifestyle risk factors were collected from the baseline survey, including age, sex and rural or urban residence. Residence status referred to regions in which the survey was conducted. Household income was categorised as less than 9999 Chinese Yuan, 10,000 to 34,999 Chinese Yuan, and more than 35,000 Chinese Yuan. Education was categorised as uneducated and primary school, middle and high school, college/university graduate and above. Occupation was categorised as agriculture workers, factory workers, clerk (i.e., professional/technical, administrator/manager, self-employed and others) and unemployed (i.e., unemployed, retired and housewife/husband). In addition, the marital status of participants was divided into married and unmarried groups. Widowed, divorced and never married were assigned to the unmarried group. Participants’ lifestyle information included systolic blood pressure (SBP), MET, body mass index (BMI), smoking habits, and alcohol consumption. SBP was recorded as the average of two measurements. The amount of daily physical activity was represented by metabolic equivalent hours per day (MET). MET was quantified based on the usual type and duration of physical activities required for work, commuting and household chores, as well as recreational exercise completed during the past 12 months. BMI was calculated using the following formula: BMI=Weight (kg)/ Height^2^ (m^2^). Smoking history and alcohol consumption were assessed by self-reported lifestyle status and classified as “frequent”, “occasional”, and “non” smoker/ drinker.

Furthermore, a series of sleep-related covariates including sleep duration, snoring, taking medication for sleep, daytime dysfunction, difficulty falling asleep and interrupted sleep were considered. The information on total sleep duration was compiled based on respondents’ self-reported answers to the question “How many hours do you typically sleep per day?” Participants could answer this question in increments of 1 h. Based on their self-reported sleep duration, participants were classified into 3 groups: <=6 h, 7–9 h, and > =10 h. The information on snoring, taking medication for sleep and daytime dysfunction was collected via the questions, “Do you snore during sleep?”, “During the past month did you require medication at least once per week to help fall asleep?” and “During the past month did you have difficulty staying alert while at work, eating or meeting people during the daytime?” In the case of snoring, participants whose answers were “Yes, usually” and “Yes, sometimes” were classified as “snoring”, while those who answered “No” were classified as “non-snoring”. Also, by asking the question “During the past month, did you take >30 minutes to fall asleep at night or wake up in the middle of the night?” information related to insomnia symptoms was collected as the covariate “difficulty falling asleep and interrupted sleep”.

In addition, information regarding self-reported disease status including cancer, hypertension, cardiovascular disease, diabetes, cirrhosis, hepatitis, stroke, renal disease, asthma, and rheumatoid arthritis was collected as covariates. People were asked, “Has a doctor ever told you that you had had the following disease?” People who answered “Yes” to a specific disease were regarded as having relevant disease history.

### Analysis

Descriptive statistics were conducted to analyse the baseline characteristics of participants, including demographic and socio-economic characteristics, lifestyle risk factors, and sleeping behaviours.

The association between daytime napping and depression was analysed using a series of logistic models. The non-napping group was chosen to be the reference group in the model. All the odds ratios were presented with 95% confidence intervals (95% CI). Total sleep duration was classified into three categories, while daytime dysfunction, snoring, taking medication for sleep and difficulty falling asleep and interrupted sleep were dichotomous variables. The basic demographic characteristics, lifestyle factors, the nine self-reported disease status, as well as sleep-related factors including daytime dysfunction, snoring, sleep duration, difficulty falling asleep and interrupted sleep and taking medicine for sleep were adjusted as confounders in all logistic regression models. Stratified analyses were undertaken to understand how age, residence, education, household income and marital status potentially influence the association between daytime napping and depression. Odds ratios were presented after adjusting for all other demographic characteristics, lifestyle risk factors, sleep-related factors, and disease history.

All analyses were conducted using SAS version 9.4 (SAS Institute, Cary, North Carolina, USA).

## Results

The basic characteristics of participants for females and males are shown in Table 1. Among all 512,891 participants included in the analysis, 59.00% (*n* = 302,632) were female. The mean age of the female and male population was 50.96 (SD = 10.48) and 52.35 (SD = 10.90), respectively.

**Table 1:** Basic characteristics of participants in females and males (*N* = 512,891)

	Female (*N* = 302,632)	Male (*N* = 210,259)
Basic characteristics		
Age, years *	50.96 (10.48)	52.35 (10.90)
Urban, %	44.55	43.45
Married, %	88.98	92.90
Education, %		
Uneducated & Primary school	56.72	42.23
High schools	38.83	49.90
University	4.45	7.87
Income (Yuan), %		
< 9999	29.77	26.04
10,000–34,999	53.78	53.73
> 35,000	16.45	20.23
Occupation, %		
Agriculture worker	40.55	43.41
Non-agriculture worker	59.45	56.59
Lifestyle factors		
SBP, mmHg *	129.87 (21.99)	132.82 (20.04)
MET, MET-hour/day *	20.42 (12.76)	20.03 (15.29)
Depression, %	0.78	0.46
Smoking, %		
Never	94.93	14.42
Occasional	2.70	24.52
Regular	2.37	61.07
Alcohol, %		
Never	63.58	20.36
Occasional	32.96	40.14
Regular	3.46	39.51
Sleep Status		
Having daytime sleep, %	18.35	24.52
Sleep hours, hours *	7.35 (1.40)	7.42 (1.33)

*Continuous variables are presented in Mean (SD)

Table 2 presents the relative odds of depression with regard to having a daytime nap, daytime dysfunction, snoring, sleep duration, taking medicine for sleep, smoking and drinking alcohol in the overall population. Within the daytime nap group, the odds ratio of depression in females was 1.15 (95% CI: 1.01–1.31), while the odds ratio for males was 1.42 (95% CI: 1.18–1.71). Having daytime dysfunction and taking medication for sleep had a strong relationship with depression in both sexes with the odds ratios varying between 2.79 (95% CI: 2.46–3.17) and 1.84 (95% CI: 1.57–2.14) in females, and 2.99 (95% CI: 2.40–3.73) and 2.00 (95% CI: 1.51–2.63) in males. Sleep duration and depression demonstrated a U-shape distribution. Compared with people who sleep 7–9 h per day, people who slept ≤6 h or ≥ 10 h per day had 1.64 (95% CI: 1.48–1.82) and 1.72 (95% CI: 1.43–2.05) times greater likelihood, respectively, of being associated with depression in the female population, and 1.70 (95% CI: 1.46–1.98) and 1.65 (95% CI: 1.28–2.12) times greater likelihood, respectively, in the male population. The relationship between snoring and depression was significant in the female population with an odds ratio of 1.34 (95% CI: 1.19–1.50).

**Table 2:** Relative odds of depression with having daytime nap and sleep-related factors in overall population (N = 512,891) *

	Female (*N* = 302,632)	P	Male (N = 210,259)	P
Have daytime nap **	1.15 (1.01–1.31)	0.0495	1.42 (1.18–1.71)	0.0002
Have daytime dysfunction	2.79 (2.46–3.17)	< 0.0001	2.99 (2.40–3.73)	< 0.0001
Have difficulty falling asleep and interrupted sleep	3.05 (2.75–3.39)	< 0.0001	3.69 (3.14–4.33)	< 0.0001
Snore during sleep	1.34 (1.19–1.50)	< 0.0001	1.17 (0.99–1.39)	0.0586
Take medicine to sleep	1.84 (1.57–2.14)	< 0.0001	2.00 (1.51–2.63)	< 0.0001
Sleep duration				
≤ 6 h	1.64 (1.48–1.82)	< 0.0001	1.70 (1.46–1.98)	< 0.0001
≥ .0001.4	1.72 (1.43–2.05)	< 0.0001	1.65 (1.28–2.12)	0.0001
Smoking				
Occasional smoke	1.25 (1.00–1.55)	0.0502	1.03 (0.82–1.30)	0.7901
Frequent smoke	1.39 (1.13–1.73)	0.0024	1.10 (0.89–1.35)	0.3906
Alcohol				
Occasional drink	1.04 (0.94–1.15)	0.4709	1.06 (0.89–1.27)	0.4913
Frequent drink	1.03 (0.82–1.29)	0.7979	0.88 (0.73–1.07)	0.2019

*Odds ratio was adjusted for residency, age, family mental disorder history, blood pressure, education, income, occupation, BMI, marital status, smoking, alcohol, MET statuses, sleep snoring, taking medicine for sleep, daytime dysfunction, difficulty falling asleep and interrupted sleep, total sleep duration, and disease statuses

**“Have no daytime nap”, “have no daytime dysfunction”, “don’t have difficulty falling asleep and interrupted sleep”, “don’t snore during sleep”, “don’t need to take medicine to sleep”, “have sleep duration of 7–9 h per day”, “never smoke”, and “never drink alcohol” groups were chosen to be the reference groups

Relative odds of depression with daytime nap stratified by confounders as listed above were presented in Table 3. A significantly positive association between daytime nap and depression was found in people 45 to 65 years of age in both genders (female: OR = 1.24, 95% CI: 1.03–1.48; male: OR = 1.41, 95% CI: 1.10–1.82). The association also existed in people 45 years old or younger. Daytime napping was significantly associated with depression among rural residents (OR = 1.31, 95% CI: 1.13–1.52) rather than urban residents (OR = 0.75, 95% CI: 0.65–0.87). In terms of education, the odds ratio of depression of those with daytime napping was 1.42 (95% CI: 1.22–1.66) among uneducated or primarily educated population. The odds ratio of depression in married population who took daytime nap was 1.14 (95% CI: 0.98–1.34) in female and 1.45 (95% CI: 1.18–1.79) in male. The association between depression and daytime napping was insignificant in unmarried population.

**Table 3:** Relative odds of depression with daytime nap stratified by residency, education, income, occupation, MET and age *

	Female (N = 302,632)	P	Male (*N* = 210,259)	P	Overall (N = 512,891)	P
Age						
< =45	1.09 (0.87–1.38)	0.4508	1.43 (1.04–1.97)	0.0279	1.23 (1.02–1.48)	0.0286
45–65	1.24 (1.03–1.48)	0.0202	1.41 (1.10–1.82)	0.0078	1.29 (1.12–1.49)	0.0006
> 65	0.90 (0.58–1.40)	0.6394	1.12 (0.65–1.94)	0.6905	1.01 (0.72–1.42)	0.9483
Residency						
Urban	0.71 (0.60–0.84)	< 0.0001	0.89 (0.63–1.17)	0.4073	0.75 (0.65–0.87)	0.0001
Rural	1.13 (0.93–1.39)	0.2168	1.66 (1.31–2.10)	< 0.0001	1.31 (1.13–1.52)	0.0003
Education						
Uneducated & Primary school	1.37 (1.14–1.65)	0.0009	1.54 (1.15–2.05)	0.0037	1.42 (1.22–1.66)	< 0.0001
High schools	0.93 (0.76–1.14)	0.4905	1.30 (0.99–1.60)	0.0551	1.06 (0.90–1.24)	0.4975
University	1.29 (0.68–2.45)	0.4309	1.68 (0.87–3.26)	0.1226	1.44 (0.92–2.25)	0.1093
Marriage						
Married	1.14 (0.98–1.34)	0.0942	1.45 (1.18–1.79)	0.0005	1.24 (1.10–1.40)	0.0007
Unmarried	1.14 (0.88–1.49)	0.3205	1.28 (0.85–1.91)	0.2304	1.22 (0.99–1.52)	0.0685
Income (Yuan)						
< 9999	1.12 (0.90–1.40)	0.0059	1.25 (0.93–1.67)	0.1357	1.16 (0.98–1.39)	0.0870
10,000–34,999	1.14 (0.94–1.38)	0.1897	1.62 (1.23–2.12)	0.0006	1.29 (1.11–1.50)	0.0011
> 35,000	1.12 (0.77–1.65)	0.3186	1.36 (0.79–2.34)	0.2758	1.15 (0.85–1.57)	0.3716

*Odds ratio was adjusted for residency, age, family mental disorder history, blood pressure, education, income, occupation, BMI, marital status, smoking, alcohol, MET statuses, sleep snoring, taking medicine for sleep, daytime dysfunction, difficulty falling asleep and interrupted sleep, total sleep duration, and disease statuses. “No daytime nap” group was chosen to be the reference group

## Discussion

Through the analysis of trends among 0.5 million Chinese citizens from 10 regions, the study’s finding suggested a positive association between daytime napping and depression in Chinese population. The association between depression and a series of sleep-related factors including daytime dysfunction, snoring, sleep duration, difficulty falling asleep and interrupted sleep and taking medication for sleep was also discovered. Further analyses indicated that socio-economic status, including residency, education background, and marital status could modify the underlying associations between daytime napping and depression. In both the female and male population, the association between daytime napping and depression was more pronounced in people who lived in rural areas, received lower-level education, and were younger than 65 years of age.

This study was based on the baseline survey of a Chinese cohort study of 0.5 million adults [51]. It was one of the few studies using a large sample to evaluate the association between daytime napping and depression in the Chinese population whose daytime nap pattern was prevalent and representative for sleeping habits in other areas of the world. The large sample size could ensure significant statistical power in identifying small difference. Also, this study clarified and expanded upon puzzling findings from prior empirical work in this area. To minimise the confounding bias in the relationship between daytime napping and depression, the present study adjusted series of confounders including lifestyle factors, sleep-related factors and depression-related diseases in the regression model. The approaches to controlling bias in daytime napping were also helpful for further studies in this field. In addition, the study provided a new angle in discussing sleep and depression. It not only examined the concept of daytime napping, but also brought attention to the potential modifying effect of socio-economic status and age.

Nonetheless, the study also had its limitations in the analysis. First, the study had limitations in sleep measurement. Without having accurate sleep measurement, the present study only assessed whether the participants had regular napping habits or not. Also, due to the lack of validated scales or experiments, information collected merely included self-reported daytime nap status, sleep duration, daytime dysfunction and difficulty falling asleep and interrupted sleep factors. Consequently, it was not possible to investigate the quantitative association between exact napping durations and depression, which could be of important scientific value. Furthermore, the healthy volunteer effect, in which the participants of the study may tend to be more interested in their health status than the general public, might attenuate the association between depression and daytime napping because of the elimination of outliers. Moreover, the cross-sectional nature of the study was insufficient to establish a causal relationship between the two factors. The present study only aimed to show the cross-sectional associations of daytime napping and depression; further prospective and experimental studies are needed to determine a causal relationship.

The study found that the prevalence rate of depression in the past 12 months was 0.78 and 0.46% in the female and male population, respectively. These prevalence rates were lower than the rates reported in other studies. Lee et al. [52] reported that the estimated prevalence of CIDI major depressive episodes in one year was 1.8% within the Chinese metropolitan population, and another study in Yunnan province identified the 12-month prevalence of depression as 1.1% (95% CI: 0.8–1.4) using the CIDI scale [53]. Difference in diagnostic tools, sample approaches, or the quality of studies might trigger the deviation of results regarding depression prevalence [52, 54]. Moreover, because of the healthy volunteer effect, participants in the present study might be healthier than the general population, physically or mentally. Another potential reason for this discrepancy might be that the present study covered an older population (30–79 years old) than the typical onset age of depression (15–30 years old) [55].

In this study, it was found that there was a significant positive association between daytime napping and depression. Only few studies provided evidence regarding association between napping and depression [28, 29, 56–59]. Foley et al. [28] and Stone et al. [59] found that an increase in regular napping was associated with self-reported or physician-diagnosed depression in 1505 and 8101 adults, respectively, in the United States. Compared with previous studies, the present study paid full attention to the control of confounding bias. Five sleep-related factors, specific lifestyle and health factors, and nine disease history factors were adjusted in the regression model, ensuring reliable results in the present study. The process of confounder adjustment also provides repeatable approaches for further study in this field.

Daytime dysfunction, snoring and shorter sleep duration were also found to have a positive association with depression. In previous studies, daytime dysfunction, which might be a symptom of excessive daytime sleepiness, was indicated as a predictor for further depressive episodes [60]. The conclusion by LaGrotte et al. [22] that as symptoms of OSA sleep disturbance and snoring have strong association with depression was supported by current study.

Previous studies generally regarded the function of daytime napping as the reflection of daytime dysfunction and OSA [22, 27, 55]. However, the association between daytime napping and depression still remained significant after controlling sleep-related factors in the present study, indicating other existing mechanisms of daytime napping. Thus, daytime napping warrants attention as an independent public health issue with regard to mental health. Despite the fact that the detailed mechanism was still unclear, potential explanations could help to better understand the association. First, since lying down on a bed was reported as a stress coping and compensating mechanism, napping was linked with increasing mental stress and declining physical functions in previous studies [61]. It is plausible that mental disorders led to tiredness during the daytime, and daytime napping was a symptom or a proxy for depression, rather than the cause. Second, harmful effects of daytime napping include disturbing circadian rhythms and changing blood serum level, both of which were also associated with depression [62–65]. In addition, drowsiness, as an antidepressant treatment-related side effect, might trigger daytime napping in the depressed population [40]. However, with less than 10% of patients received antidepressant treatment, the effect of antidepressant drugs on the present results was minimal among Chinese population because of the high cost of imported drugs and insufficient knowledge of depression [20].

The present study also discovered a modifying effect of socio-economic status and age to such an association. Although the associations between lower SES and mental disorders have been well established in previous studies, the modifying effect of SES on the nap-depression association is still not settled [44, 66]. Because daytime napping has been suggested as a compensation for fatigue or sleep disturbance, lower SES may trigger night sleep disturbance or fatigue and aggravate the frequency or the duration of daytime napping [67]. In an impoverished population with less education or living in rural areas, “sleep disparities” defined as poor sleep quality may exert a stronger effect on mental health [68]. For example, it has been shown that shift workers of lower SES tend to experience more frequent night sleep disturbance and greater fatigue [69].

The present study also found that the association between daytime napping and depression was significant only in people less than 65 years old. Age has been considered as one of the most important factors associated with napping because nap behaviours shift through the lifespan. Previous studies have identified that the number of naps per day and the frequency of taking naps per week increases with age [70, 71]. The modifying effect of younger age to the association could possibly reflect the different patterns and quality of daytime napping between younger adults and elderly adults. Milner and Cote et al. found that older adults might need a longer nap to accrue the same benefits as younger adults because sleep is typically less restorative with advancing age [72]. Moreover, some specific modes of daytime napping in elderly people may be associated with the improvement of nighttime sleep quality, the promotion of mental health and physical well-being [73]. For example, the combination of a 30-min afternoon nap and moderately intense exercise in the evening for elderly people or the frequent napping of elderly hospitalised patients was found to ameliorate mental and cognitive functions [10, 74]. Since the mechanism among daytime napping, depression, age and SES remains unclear, definitive and prospective evidences on nap-depression association in various populations are needed to establish a causal relationship.

## Conclusions

The positive association between daytime napping and depression was one of the major findings in the study population. Both shorter and longer sleep duration, having daytime dysfunction, having difficulty falling asleep and interrupted sleep, snoring, and taking medication to fall asleep were also identified as having strong relationships with depression. Further analyses suggested that socio-economic status, including rural residency and lower educational background, could possibly modify the underlying associations between daytime napping and depression. In addition, in both female and male populations, the association was only significant in people younger than 65 years of age. There still remains a great need for additional research regarding napping, depression, and the modifying effects of age and SES in further clinical or population studies to facilitate the diagnosis and prevention of depression.

## Abbreviations

BMI: Body mass index
CI: Confidence interval
CIDI-SF: Composite International Diagnostic Inventory-Short From
EDS: Excessive daytime sleepiness
MD: Major Depressive
MET: Metabolic equivalent
OR: Odds ratio
OSA: Obstructive sleep apnea
SBP: Systolic blood pressure
WHO: World Health Organization
